# Mammalian Metallothionein-2A and Oxidative Stress

**DOI:** 10.3390/ijms17091483

**Published:** 2016-09-06

**Authors:** Xue-Bin Ling, Hong-Wei Wei, Jun Wang, Yue-Qiong Kong, Yu-You Wu, Jun-Li Guo, Tian-Fa Li, Ji-Ke Li

**Affiliations:** Department of Cardiovascular Institute, Affiliated Hospital of Hainan Medical College, Haikou 570102, China; xuebin192006@163.com (X.-B.L.); aida1860@163.com (H.-W.W.); doctorwangjun@126.com (J.W.); kongyueqiong1@medmail.com.cn (Y.-Q.K.); wuyuyou76@163.com (Y.-Y.W.); guojl79@163.com (J.-L.G.)

**Keywords:** metallothionein-2A, oxidative stress, mitogen-activated protein kinases, reactive oxygen species

## Abstract

Mammalian metallothionein-2A (MT2A) has received considerable attention in recent years due to its crucial pathophysiological role in anti-oxidant, anti-apoptosis, detoxification and anti-inflammation. For many years, most studies evaluating the effects of MT2A have focused on reactive oxygen species (ROS), as second messengers that lead to oxidative stress injury of cells and tissues. Recent studies have highlighted that oxidative stress could activate mitogen-activated protein kinases (MAPKs), and MT2A, as a mediator of MAPKs, to regulate the pathogenesis of various diseases. However, the molecule mechanism of MT2A remains elusive. A deeper understanding of the functional, biochemical and molecular characteristics of MT2A would be identified, in order to bring new opportunities for oxidative stress therapy.

## 1. Introduction

**Figure 1:**
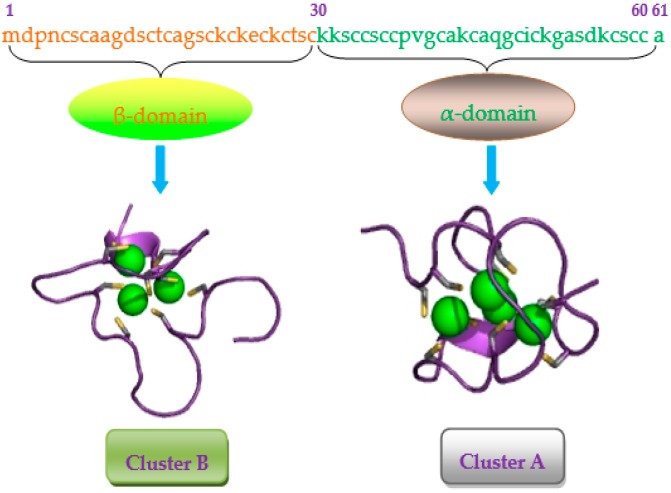
Structure of human metallothionein-2 (MT-2). Amino acid sequence and 3D structure were retrieved from UniProt (P02795). Class I MT2 contains 2 metal-binding domains: four divalent ions are chelated within cluster **A** of the α-domain and are coordinated via cysteinyl thiolate bridges to 11 cysteine ligands. Cluster **B**, the corresponding region within the β-domain, can ligate three divalent ions to 9 cysteines. The 3D structures show that cluster A could bind three metal ions (Cd^2+^), and cluster B could bind four metal ions (Cd^2+^), respectively.

Mammalian metallothioneins (MTs) are low molecular mass (6–7 kDa) proteins, which become a big family of metal-binding and metal-absorbing, cysteine-rich molecules [1,2]. Human MTs consist of 11 functional isoforms: MT-1 (A, B, E, F, G, H, M, and X), MT-2 (known as MT2A), MT-3, and MT-4, and furthermore both MT-1 and MT2A are expressed in various organs, tissues, and cultured cells, while MT-3 is expressed mainly in the brain and MT-4 most abundant in certain epithelial tissues [3]. Frankly, although MT-1 and MT2A have been largely studied together, MT2A have specific functions in regulating autophagy and apoptosis [4], and increasing risk of prostate cancer [5], as well as ductal breast cancer [6]. MT2A is composed of 61 amino acids and characterized by low molecular weight (7kDa), high cysteine content(30%) and lack of aromatic amino residues [7]. The binding metal of apoMT2A could form α-domain and β-domain, promoting convergence to the dumbbell-shaped conformation [8] (Figure 1). The function of MT2A is to regulate metal homeostasis, detoxification, oxidative stress, immune defense, cell cycle progression, cell proliferation and differentiation, and angiogenesis [9,10,11,12,13]. Importantly, MT2A could regulate MAPKs and play a crucial pathophysiological role in anti-oxidation, anti-apoptosis, anti-inflammation [14,15,16,17]. Reactive oxygen species (ROS) are a class of chemically reactive metabolites including superoxide anion (O_2_^•−^), hydroxyl radical (^•^OH), peroxynitrite (ONOO^−^) and hydrogen peroxide (H_2_O_2_), which could cause protein dysfunction and DNA damage, leading to gene mutations and cell death [18,19]. In recent years, ROS have generally been described as second messengers because of cellular signaling cascades and pathophysiological processes, such as proliferation, gene expression, adhesion, differentiation, senescence, apoptosis and necrosis [20,21], which are mediated by activated MAPKs [22,23,24,25]. Massive studies have revealed that oxidative stress played an important role in the pathogenesis of various diseases, such as coronary heart diseases (CHDs) [26,27,28], neurodegenerative disorders [29,30], cancer [31,32,33], and aging [34,35]. Overall, more evidences have indicated that MT2A, as a free radical scavenger, might protect cells and tissues from oxidative stress. Admittedly, present review mainly focuses on total MT rather than its subtypes, but we can presume that MT2A possibly has similar function. Consequently, this review summarizes the relationship between MT2A and oxidative stress.

## 2. Metallothionein-2A (*MT2A*) Gene Expression Regulation

MT2A could be influenced by Zn in human lymphocytes [36]. It is up-regulated during oxidative stress and hypoxia/reoxygenation (H/R) with the increasing levels of ROS [37], H_2_O_2_ [38], and various metal ions, such as Cd^2+^ and Cu^+^ [39]. MT2A promoter region contains metal responsive elements, glucocorticoid responsive elements, antioxidant responsive elements, cAMP responsive elements, tissue plasminogen activator-responsive elements and interferon responsive elements. The core promoter region of MT2A contains single nucleotide polymorphism, with 87% homozygote typical (AA) and 12.3% heterozygote (AG) [40]. MT2A is severely suppressed by knockdown of metal responsive element-binding transcription factor-1 [41].

## 3. MT2A Function

### 3.1. Anti-Oxidative Stress Injury

#### 3.1.1. The Role of Zn

MT2A is a powerful scavenger of free radicals through its cysteine residues [15]. It is a Zn chelator when the amount of Zn is excessive and as a scavenger of ROS when oxidative stress is elevated [37]. The binding Zn of apoMT would maintain the stability of MT [42], and oxidative stress could also trigger Zn to integrate with apoMT [43]. Unfortunately, no further studies focusing on MT2A have been performed. Low dose of Zn (50 μM ZnSO_4_) could up-regulate MT2A expression in reducing cytotoxicity through inhibiting oxidative stress and DNA damage [44], whereas the high dose (100 μM ZnSO_4_) is responsible for neurotoxicity through ERK1/2 [45]. MT2A is capable of binding Zn^2+^, known as Zn_7_MT2A, which affects ionic homeostasis and subsequent neurotoxicity of cultured cortical neurons [10]. The characterization of the metal-binding abilities of MT2A shows a clear preference towards Zn^2+^ coordination, compared to Cd^2+^ and Cu^+^ [39].

#### 3.1.2. MT2A and Other Antioxidants

Intracellular antioxidants commonly include glutathione (GSH), heme oxygenase-1 (HO-1), superoxide dismutase-1 and triphosphopyridine nucleotide (NAPDH) [46]. MT2A could create a new pool of thiol in cell cytosol which could attenuate the damaging effect of GSH depletors [47]. The ability of MT2A to scavenge free ^•^OH and peroxyl radicals is found to be 100-fold higher than that of GSH [48]. Both MT2A and HO-1 are increased along with ROS during oxidative stress [49]. Moreover, MT-1/2 double knockout cells would adapt to the expression of HO-1 [50]. Additionally, MT could mediate phosphorylate extracellular signal-regulated kinases (ERK), and control ROS through regulating HO-1 [51].

### 3.2. Anti-Apoptosis

A wide range of adverse stimuli, such as oxidative stress could cause cell apoptosis [21]. MT2A reduces adriamycin-induced myocardial injury through inhibition of oxidative stress-mediated mitochondrial cytochrome-c release and activated caspase-3 [47], protects human umbilical vein endothelial cells from lipopolysaccharide (LPS)-associated apoptosis, and also influences cellular behaviors such as proliferation and chemotaxis by binding to membrane receptors [52]. MT2A could also protect endoplasmic reticulum (ER) stress-induced cardiac failure associated with attenuation of myocardial apoptosis [53]. Knockdown of MT2A could down-regulate Zn level and affect cell apoptosis [4]. Moreover, MT2A is a protective protein from apoptosis by down-regulating the expression of Bax, caspase-3, caspase-9, and caspase-12 [4,54].

### 3.3. Anti-Inflammation

MT2A could regulate cell inflammatory response through inhibition of nuclear factor-κB (NF-κB) [55], and endothelial-overexpressed LPS-associated factor-1 (EOLA1) [56]. Inflammatory cytokines are released by oxidative stress [57], whereas MT2A could inhibit the activation of pro-inflammatory cytokines, such as IL-6, IL-12 and TNF-α [15]. MT-1/2 knockout would significantly aggravate renal oxidative damage and inflammation induced by intermittent hypoxiavia Nrf2 signaling pathway [58].

## 4. MT2A and Oxidative Stress

### 4.1. Subcellular Changes

#### 4.1.1. Mitochondrial Stress

**Figure 2:**
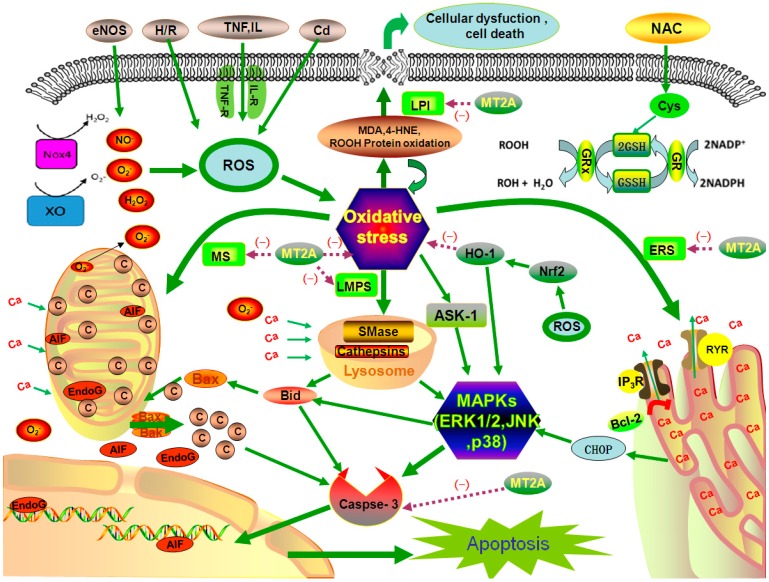
Interactions of subcellular structure during oxidative stress and activation cell apoptosis pathway through mitogen-activated protein kinases (MAPKs). Mammalian metallothionein-2A (MT2A) might inhibit oxidative stress through four subcellular structures. Mitochondrial Stress—MS, Endoplasmic Reticulum Stress—ERS, Lysosomal Membrane Permeabilization Stress—LMPS, Lipid Peroxidation Injury—LPI, Endothelial Nitric Oxide Synthase—eNOS: (green arrows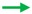: caused definitely, brown dotted arrows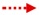: caused indefinitely, Inhibit(−)).

Oxidative stress-mediated damage to mitochondrial DNA could be observed in patients with diabetes mellitus and atherosclerosis [59]. MT2A exerts antioxidant effects against mitochondrial superoxide [60]. Over expression of MT2A can decrease oxygen consumption, down-regulate cellular ATP levels and decrease oxidative phosphorylation capacity, and interact with mitochondrial complexes indirectly, which might be involved in the inhibition of certain respiratory enzymes via metal binding [61]. MT2A could suppress ischemia/reperfusion (I/R)-induced myocardial apoptosis mediated by mitochondrial stress [62] (Figure 2). As for downstream signaling, intrinsic apoptotic signaling leads to mitochondrial membrane permeabilization and releases cytochrome-c into the cytosol through JNK signal [63].

#### 4.1.2. ER Stress (ERS)

ERS stimulates autophagy in a JNK-dependent manner and promotes cell survival during oxidative stress [64]. MT2A could suppress the expression of CCAAT/enhancer-binding protein (C/EBP) homologous protein (CHOP) during Ang II-induced ERS [65] (Figure 2). ERS augments left ventricular diameter, suppresses heart contractility, and induces liver injury, which are significantly attenuated or ablated by MTs [66,67]. Additionally, ERS leads to accumulation of unfolded proteins in ER, which could activate multiple signaling pathways including JNK, p38 and NF-κB [68,69].

#### 4.1.3. Lysosomal Membrane Permeabilization (LMP) Stress (LMPS)

Oxidative stress could induce LMP through activation of lysosomal hydrolytic enzymes [70], which causes apoptosis [71]. MT-1/2A up-regulation has been reported to protect against LMP induced by various kinds of oxidative stress [72]. Lysosomal delivery of up-regulated MT2A is the key mechanism by which autophagy protects cells against LMPS [73] (Figure 2). LMPS is associated with activation of MAPKs, for instance, JNK has an important pro-apoptotic function, which mediates the upstream of LMP and phosphorylation [63].

#### 4.1.4. Biological Membrane Lipid Peroxidation Injury (LPI)

MT2A could inhibit LPI and improve recovery after transient brain I/R in rats [74] and other researches have demonstrated that LPI increased by I/R-induced myocardial injury are dramatically decreased in MT-overexpressing mice and the oxidative damage in the lipid membranes is related to lipid peroxide (LPO)and MT levels [62] (Figure 2).

### 4.2. The Role of MT2A in MAPKs Signals

**Figure 3:**
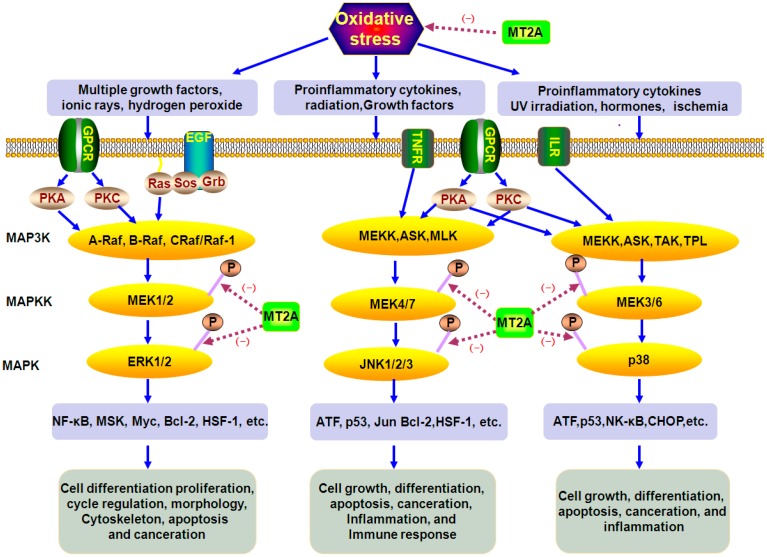
MAPKs pathway during oxidative stress. MAPKs pathway is typically initiated by G protein–coupled receptor or by stress stimuli, then triggers a cascade of phosphorylation reactions, and finally leads to various cell biological effects. MT2A might inhabit MAPKs in oxidative stress. (blue arrows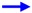: caused definitely, brown dotted arrows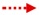: caused indefinitely, Inhibit(−).)

MAPKs comprise a family of serine/threonine phosphorylating proteins, which contain three main branches: ERK, JNK and p38 [75]. MTs play an important role in improving the LPS-induced cardiac dysfunction with activated MAPK [76] (Figure 3). However, MT plays a key role in preventing hypoxia-induced renal injury via Nrf2, owing to inactivation of AKT and ERK [58]. Arsenic trioxide could induce H9c2 cell death in a dose- and time-dependent manner with a significant activation of MAPKs, but not in MT-H9c2 cells [77]. Importantly, the protective effect of MT on arsenic trioxide-induced apoptotic cell is completely recaptured in heart with a significant prevention of MAPKs [77]. In Cd-induced apoptotic cells, MT is less expressed in Cd-sensitive cells but p-JNK is increased, and a strong activator of JNK, R0318220, could reverse the Cd-sensitive phenotype in Cd-resist cells, and this research also showed that p-JNK1/2 is markedly up-regulated in MT^−/−^cells compared with MT^+/+^cells through Cd treatment, suggesting that MT might inhibit JNK1/2 activation [16]. Another study has indicated that the suppression of JNK is mediated by ROS [78]. Whether MT directly inhibits JNK phosphorylation or not remains elusive in present studies.

## 5. MT2A and Disease 

### 5.1. Cardiovascular Disease

MT2A is a potent antioxidant in heart [37,53,79] (Table 1). More importantly, antioxidant is shown to exert beneficial effects in hypertension, atherosclerosis, ischemic heart disease, cardiomyopathy and congestive heart failure [17,80]. Although the mechanism underlying myocardial protection from I/R injury through MT has not been fully understood, a large pool of evidence has demonstrated that oxidative stress is a critical mediator for myocardial damage during I/R [27,62]. MT2A might play a role in cardiovascular protection through radical scavenging activities and suppression of lipid peroxidation [81]. MT2A polymorphism is associated with atherosclerosis on coronary artery [26], and carotid artery [82]. The cardioprotective property of MT is involved in diabetes mellitus-, obesity- and aging-induced cardiac damage [28,83]. ERS directly triggers cardiomyocyte dysfunction and MT could ablate the process through up-regulating the level of JNK phosphorylation [84]. ERS inhibitor tauroursodeoxycholic acid could reverse the process [85]. Moreover, MT is able to prevent myocardial anomalies through restoration of autophagy in hypertensive heart diseases [79].

### 5.2. Nervous System Disease

Multiple nervous system diseases are closely related to MT2A. MT2A is the most significantly up-regulated transcript in ischemic head [86], and it is a novel neuroprotective factor to prevent ischemic injury [74] (Table 1). Simultaneously, MT2A is a critical component in the maintenance of immune homeostasis, as it is demonstrated in autoimmune encephalomyelitis disease [87]. Parkinson’s disease is one of the most common progressive neurodegenerative disorders with increased oxidative stress and MT2A released from astrocytes is a potent protector of dopaminergic neuron [88]. Alzheimer’s disease is triggered by the deposition of insoluble extracellular b-amyloid (Ab) plaque, and MT2A is capable of protecting against Ab aggregation and toxicity for therapeutic approach to Alzheimer’s disease [10]. Additionally, MT-1/2 knockout mice would result in embryonic lethality in a model of Menkes disease (a copper efflux disease) [89].

### 5.3. Cancers

MT-1/2 deficiency predisposes mice more sensitive to early life Pb exposure with regard to testes tumors, renal and urinary bladder preneoplastic lesions [33]. MT2A over-expression is associated with cell proliferation in cancerous breast tissue [90], with significantly modified breast cancer risk, and cell cycle is inhibited through silencing MT2A [91]. MT2A predicts high therapeutic value in hepatocellular carcinoma [92], whereas predicts poor survival in glioblastoma multiforme [93]. MT2A might be a chemosensitive indicator in gastric cancer (GC) [94], and another study found that MT2A might play a role in suppressing tumor activity through inhibiting NF-κB and might be a prognostic biomarker and potential target for individual therapy of GC [95] (Table 1). MT2A polymorphismis closely correlated with neoplasm, implicated in laryngeal cancer [96]. In addition, MT2A contributes to chemotherapy resistance in osteosarcoma [97]. Finally, it has to be mentioned that the MT2A has differential outcome in various types of cancer that may be tissue or cell type dependent, just like E2 factor, including cellular proliferation, apoptosis and tumor kinetics [98].

### 5.4. Aging

MT2A is crucial for the immune efficiency during aging and age-related diseases [99]. Up-regulated expression of MT2A in kidney with aging might play a protective role, which is closely related to aging [34]. Additionally, MT2A gene polymorphisms are associated with aging in Turkey [100] (Table 1).

### 5.5. Diabetes Mellitus

MT2A could prevent diabetes-induced cardiac ERS, which contributes to prevent dilated cardiomyopathy (DCM) [65] (Table 1). MT2A plays an important role in antioxidant defense in type2 diabetes mellitus through modulating glutathione, which promotes phosphorylation of insulin receptors through enhancing transportation of glucose into cells [101]. Moreover, MT2Ais correlated to diabetic atherosclerosis in patients [102].

### 5.6. Hepatal and Gastrointestinal Diseases

It was found that MT2A is slightly more expressed in both chronic hepatitis and Wilson’s disease [103] (Table 1). Furthermore, MT2A could activate human hepatic stellate cells to up-regulate the expression of collagenase genes, which might exert the therapeutic effect upon liver fibrosis [104]. Additionally, MT2A could prevent colonic mucosal inflammation in murine experimental colitis [105].

## 6. Conclusions

MT2A is intimately associated with oxidative stress, mediated by subcellular pathways of mitochondria, ER, lysosomal, and lipidosome, as well as MAPKs (ERK, JNK and p38) signals.

## Figures and Tables

**Table 1:** Summary of MT2A in relation to disease.

Reference	Tissue Type/Sample Size	Findings
Chung, et al. [10]	Rat: cortical neuron cells;treated with/without Aβ_1–40_; *n* = not disclosed	MT-2A was capable of therapeutic approach to AD
Yang, et al. [26]	Human: Peripheral blood; 287CHD; 226 control	The gene polymorphism of MT2A-838G/C was correlated to CHD
Xu, et al. [65]	Mice: myocytes; 6 wild-type; 6 cardiac-specific MT transgenic mice; Rats: H9c2 and H9c2MT7 cells; *n* = not disclosed	MT2A could prevent diabetes-induced cardiac ERS, which contributed to prevent DCM
Xue, et al. [37]	Rats: H9c2 and H9c2MT7 cells; *n* = not disclosed	MT2A markedly increased oxidative protection induced by H/R or Cd toxicity in rat cardiac myocytes
Jakovac, et al. [87]	Rats: Tissues: spinal cord, liver; BBH and DA	MT2A had neuroprotective role of autoimmune encephalomyelitis
Miyazaki, et al. [88]	Mice: Tissues: astrocytes, the striatum; 6-hydroxydopamine-Lesioned parkinsonian model mice; control; *n* = not disclosed	MT2A provided a promising therapeutic strategy in Parkinson’s disease
Pan, et al. [95]	Human: Gastric tumor tissue; 684 GCs patients cohort; 258 GC patients subset	MT2A might be a chemosensitivity indicator in GC patients
Kayaalti, et al. [100]	Human: Peripheral blood; 354 individuals aged between 18 and 95	The IL-6-174C+ carriers and MT2A-5 G-carriers might be more advantageous for longevity
Giacconi, et al. [102]	Human: Peripheral blood; 91 Type 2 diabetes patients; 188 control	The MT2A polymorphism was associated with Type 2 diabetes and atherosclerosis
Nakazato, et al. [103]	Human: Peripheral blood; 18 chronic hepatitis C patients and 19 Wilson’s disease patients; 200 control	A significantly elevated MT2A was found in patients with chronic hepatitis and Wilson’s disease
Xu, et al. [104]	Human: LX-2 cell from human hepatic stellate; pEGFP-N1-hMT-IIA and pEGFP-N1 were transfected into LX-2 cells; *n* = not disclosed	Liver fibrosis might be treated by MT2A

MT7: human MT-IIA over-expressing cardiac cell line; BBH: bovine brain homogenate rats; DA: Dark Agouti rats; IID: itai-itai disease; DCM: dilated cardiomyopathy.
